# Association between intensity of STI screening and development of antimicrobial resistance in *N. gonorrhoeae* in 12 cities in the USA: An ecological study

**DOI:** 10.12688/f1000research.15569.4

**Published:** 2018-10-31

**Authors:** Chris R. Kenyon

**Affiliations:** 1 Department of Clinical Sciences, Institute of Tropical Medicine, Antwerp, 2000, Belgium; 2 Department of Medicine, University of Cape Town, Cape Town, South Africa

**Keywords:** N. gonorrhoeae; STI screening; antimicrobial resistance; MSM

## Abstract

In this study, we assessed if there was a city-level association between sexually transmitted infection (STI) screening intensity in men who have sex with men and antimicrobial sensitivity in *Neisseria gonorrhoeae* in the United States, 2007 to 2013.  We found positive associations between STI screening intensity and increases in minimum inhibitory concentrations for certain antimicrobials. Not all positive associations were statistically significant and the associations found to be statistically significant varied between the different analyses. Further studies are therefore required to assess if there is a causal relationship between the intensity of STI screening in MSM and gonococcal resistance.

## Introduction

In the United States (USA) the prevalence of antimicrobial resistance in *Neisseria gonorrhoeae* has typically been higher in men who have sex with men (MSM) than men who have sex with women (MSW) and women ^1, 2^. It has also frequently been noted to be highest in the West and lowest in the South ^1– 3^. Resistance has characteristically emerged in the West Coast and Hawaii and then spread eastward ^1– 3^. This patterning of spread has led to the view that a primary driver of resistance is the import of resistant gonococci from eastern Asia and other world regions ^3^. In support of this theory, a number of studies have documented travel as a means of import of resistance in the USA ^4, 5^. A systematic review of risk factors associated with resistance in *N. gonorrhoeae,* however, found that a history of sex with partners abroad was associated with resistance in 6 studies and was not associated with resistance in 7 studies ^6^. Furthermore, the evidence that travel plays a seminal role in the emergence of resistance in MSM is not that compelling. An analysis of data from the Gonococcal Isolate Surveillance Project (GISP) 2002 to 2007, for example, found a pronounced increase in ciprofloxacin-resistance in MSM and a smaller and later increase in MSW; the association with recent travel was negative in MSM and borderline positive in MSW ^7^.

Antimicrobial resistance results largely from exposure to antimicrobials ^8, 9^. This has been extensively documented *in vitro* and *in vivo* but for various reasons antimicrobial pressure at a population level may be more important than at an individual level in determining risk of development of antimicrobial resistance ^8, 10, 11^. In the case of *N. gonorrhoeae,* extensive antimicrobial exposure in a population would be predicted to result in a high prevalence of resistance genes in the pharyngeal microbiomes that could then be taken up (via transformation) by *N. gonorrhoeae* and thereby provide it with a fitness conferring resistant phenotype in the setting of ongoing high antimicrobial consumption ^12^. These insights have provided the rationale for ecological level studies that have generally found strong associations between the intensity of antimicrobial use and the prevalence of resistance to that antimicrobial ^8, 13^. A recent study from the USA however found no association between an increase in *N. gonorrhoeae* minimum inhibitory concentration (MIC) for azithromycin, ceftriaxone, cefixime and ciprofloxacin in the 23 GISP sites and the consumption of antimicrobials in the surrounding county ^3^. A weakness of this study design was the use of total-consumption-of-antimicrobials by the entire county population as the explanatory variable. Since resistance has repeatedly emerged in certain MSM populations, it would be prudent to assess if this emergence is correlated with antimicrobial consumption in this group rather than the entire population. One major driver of antimicrobial consumptions in MSM is sexually transmitted infection (STI) screening. Because most *N. gonorrhoeae* and *Chlamydia trachomatis* in MSM are carried asymptomatically in the anorectum and oropharynx, screening for these STIs may result in a large increase in antimicrobial exposure. A modeling study for example found that increasing annual gonorrhea/chlamydia screening in an MSM population from 3 to 50% would result in a 11-fold increase in antimicrobial exposure ^14^. In this exploratory paper we hypothesized that the intensity of STI testing plays a role in the genesis of resistance in MSM via the associated increase in antibiotic exposure.

## Methods

We assessed if there was an ecological-city-level-association between the intensity of STI testing in MSM in the USA and the development of antimicrobial resistance in *N. gonorrhoeae*.

Data for STI screening was taken from the 2005, 2008 and 2011 National HIV Behavioral Surveillance MSM (NHBS-MSM) studies. These cross-sectional surveys done in 21 cities asked respondents about STI testing in the preceding 12 months. The 2005 survey (n=10,030) asked if respondents had been tested for syphilis/gonorrhea/another-STI during the preceding 12 months (single question), the 2008 survey (n=8,175) if they had been tested for syphilis in the preceding 12 months, and the 2011 survey (n=8,012) if they had been tested for gonorrhea, chlamydia or syphilis in the previous 12 months (3 questions).

Data for the change in city geometric mean *N. gonorrhoeae* MIC between 2005 and 2013 was taken from GISP data ^3^. The geometric mean MIC was calculated as the *n*th root of the product of *n* MIC values. Spearman’s correlation was used to assess if there was an association between (1) the prevalence of STI testing in each survey and the increase in geometric mean MIC of cefixime, ceftriaxone and azithromycin in *N. gonorrhoeae* between 2005 and 2013 and (2) the percent reporting screening for any STI in the 2011 survey and geometric mean MIC for the three antimicrobials in the following year. These three antibiotics were chosen since these were the recommended antibiotics for *N. gonorrhoeae* therapy since 2007 ^1^. All analyses were conducted in STATA 13.

## Results

**Figure 1.**
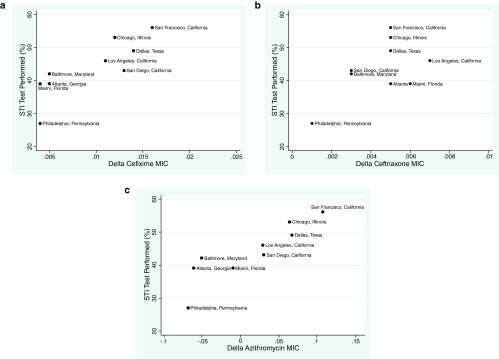
Scatter plots of change in the minimum inhibitory concentrations (MICs) between 2005 and 2013 and the percent of respondents reporting in 2005 that they had a bacterial STI test in the prior 12 months for ( **a**) cefixime, ( **b**) ceftriaxone and ( **c**) azithromycin by USA city (data sources detailed in *Methods*).

Twelve cities participated in both the NHBS-MSM and GISP surveys (n=9 for 2005, n=12 for 2008, n=12 for 2011). The intensity of self-reported STI testing in 2005 varied between 27% and 56% (median 43%, IQR 39–49). There was little change in the relative positions of the cities in terms of testing intensity between 2005 and 2008 (rho=0.87, p=0.002) and 2005 to 2011 (rho=0.81, p=0.008). Cities in the West tended to have higher STI testing rates than cities in the South ( Figure 1). In 2011, the percent reporting testing for gonorrhea was strongly correlated with the percent reporting testing for chlamydia (rho=0.99, p<0.001) and syphilis (rho=0.98, p<0.001). In general, the *N. gonorrhoeae* geometric mean MIC for cefixime and azithromycin increased more rapidly than ceftriaxone in all cities (data not shown).

In 2005, significant positive associations were found between STI screening and the increase in MIC (2005 to 2013) of cefixime (rho=0.88, p=0.002), azithromycin (rho=0.93, p<0.001) but not ceftriaxone (rho=0.27, p=0.491; Figure 1). Likewise in 2008, there was a positive correlation between the percent reporting testing for syphilis in the prior 12 months and increase in MIC of cefixime (rho=0.71, p=0.010), azithromycin (rho=0.791, p=0.002) but not ceftriaxone (rho=0.36, p=0.247). A positive association was also found for the percent reporting testing for gonorrhea in 2011 and an increase in MIC for cefixime (rho=0.63, p=0.026) and azithromycin (rho=0.64, p=0.024) but not ceftriaxone (rho=0.56, p=0.062). The results for chlamydia and syphilis testing were similar (data not shown).

Spearman’s correlation between percent reporting screening for any STI in 2011 and geometric mean MIC for the three antimicrobials in the following year revealed a positive association for ceftriaxone (rho=0.64, p=0.026) but not for azithromycin (rho=0.45, p=0.141) or cefixime (rho=0.31, p=0.325).

## Discussion

There was a roughly two-fold variation in the proportion of MSM in different cities reporting testing for bacterial STIs. The proportion testing for bacterial STIs was associated with an increase of MIC for cefixime and azithromycin but not ceftriaxone over the time period 2005 to 2013. The correlations between percent screening in 2011 and MIC in the following year were different in that the only significant association was for ceftriaxone. Of note all six correlations between percent screening and MIC were positive. The difference between the two types of analyses related to the strengths of the associations.

A plausible reason for the lack of association between ceftriaxone and MIC change (2005–2013) is that ceftriaxone has been used almost exclusively in combination with azithromycin ^3, 15^ and even on its own may be less susceptible to the develop of resistance than cefixime and azithromycin ^16^. These findings are compatible with the theory that screening intensity plays a role in the selection of antimicrobial resistance in *N. gonorrhoeae* in MSM. Alternatively they could reflect more intense screening in sites where there is more concern about antimicrobial resistance.

The findings should however be regarded as tentative due to a number of methodological weaknesses: the sample size was small the outcome variable (increase in geometric MIC) referred to all men sampled in GISP and not just MSM, the explanatory variable was only evaluated at three time points, the explanatory variable measured ‘STI testing’ and not ‘STI screening’ and possible confounders were not controlled for. Increased testing could, for example, be associated with other factors that may be associated with antimicrobial resistance such as greater risk behavior, more frequent travel, HIV-infection and access to medical care. Likewise we did not control for changes over time in the percent of GISP samples derived from MSM which may have influenced the geometric mean MICs. Finally, the decline in the absolute number of participants in the NHBS surveys over time and variations in participation rates by city could introduce biases.

Future studies that wish to evaluate the screening-resistance hypothesis could assess if there is an association between bacterial STI screening intensity and resistance in *N. gonorrhoeae* in bigger samples in the USA or elsewhere. Testing this hypothesis in Europe would be instructive since the proportion of MSM reporting anal screening for bacterial STIs in the prior 12 months in 38 different European countries ranges from 9.1% in Romania to 79.6% in Malta (median 18.5%, IQR 13.5–28.4) ^17^. Furthermore whilst two of these countries that report high STI screening rates in MSM (the United Kingdom ^17^ and the Netherlands ^17^) have found an association between resistance in *N. gonorrhoeae* and MSM ^18, 19^, other countries in Europe have not found this association ^15^.

The recent emergence of combined azithromycin/ceftriaxone resistant *N. gonorrhoeae* provides additional motivation to better characterize the underlying determinants of the differential emergence of resistance in MSM and other populations ^20^. If screening intensity is found to play a role then this could be taken into account in development of an optimal STI screening strategy. 

## Data availability

Data for STI screening: National HIV Behavioral Surveillance MSM (NHBS-MSM) studies

GISP data: Technical appendix of 3
