# Supplementary material for: Association between intensity of STI screening and development of antimicrobial resistance in N. gonorrhoeae in 12 cities in the USA: An ecological study
Source: F1000Res. 2018 Oct 31;7:1237. Originally published 2018 Aug 10. [Version 4] doi: 10.12688/f1000research.15569.4 (PMC6213784; doi:10.12688/f1000research.15569.4)
Supplement: Peer review history

# Peer review: Referee response for version 4

**DOI:** 10.5256/f1000research.18434.r40078

**Published:** 2018-11-01

**Authors:** Edward Goldstein

**Affiliations:** 1 Center for Communicable Disease Dynamics (CCDD), Department of Epidemiology, Harvard T.H. Chan School of Public Health, Boston, MA, USA

The reviewer wishes to thank the author for the edits introduced in the most recent revision. It might be helpful if the names of antimicrobials considered in the analysis were listed in the Abstract. Also, the sentence "Not all positive associations were statistically significant" probably needs editing as the term "positive associations" usually implies that they are significant. Perhaps the penultimate sentence in the Abstract could be replaced by something like "Several types of analyses relating STI screening intensity and changes in minimal inhibitory concentrations were performed, and the associations found to be statistically significant varied between the different analyses."

I have read this submission. I believe that I have an appropriate level of expertise to confirm that it is of an acceptable scientific standard.

# Peer review: Referee response for version 3

**DOI:** 10.5256/f1000research.18232.r39543

**Published:** 2018-10-22

**Authors:** Edward Goldstein

**Affiliations:** 1 Center for Communicable Disease Dynamics (CCDD), Department of Epidemiology, Harvard T.H. Chan School of Public Health, Boston, MA, USA

The reviewer wishes to thank the author for revising the submission.

 In the most recent submission, the author chose to mostly report those findings in the Results section that show the effect of screening for azithromycin and cefixime MICs, but not ceftriaxone MICs. In the earlier response to the reviewer queries, the author wrote:

 “we have assessed Spearman's correlation between percent reporting screening for any STI in the previous 12 months and MIC for the three antimicrobials in the following year (2012):

 Azithromycin: Rho 0.45; P=0.141

 Cefixime: Rho 0.31; P=0.325

 Ceftriaxone: Rho=0.64; P=0.026”

 “We also assessed Spearman's correlation between percent reporting screening for any STI in the previous 12 months (2011 survey) and the fold change in geometric mean MIC for the three antimicrobials between

 a) 2005 and 2013:

 Azithromycin: Rho 0.77; P=0.003

 Cefixime: Rho 0.82; P=0.001

 Ceftriaxone: Rho=0.61; P=0.034”

 "b) 2009 and 2013:

 Azithromycin: Rho 0.33; P=0.299

 Cefixime: Rho -0.50; P=0.100

 Ceftriaxone: Rho=0.58; P=0.047"

 The reviewer thinks that choosing to focus on those results that stress the effect for azithromycin and cefixime MICs, but not ceftriaxone MICs could be viewed as tendentious, trying to exhibit the effect of screening on MICs for the antimicrobial that is currently used in the treatment of gonorrhea. The reviewer would suggest to give a more balanced account of the results in light of all the findings.

I have read this submission. I believe that I have an appropriate level of expertise to state that I do not consider it to be of an acceptable scientific standard, for reasons outlined above.

# Response

**Published:** 2018-10-26

**Authors:** Chris Kenyon

**Affiliations:** Institute of Tropical Medicine, Antwerp, Belgium

Thank you for pointing this out. I agree that the abstract could be construed to exaggerate the link between screening intensity and antimicrobial resistance. I have therefore changed the abstract to the following:

 We found positive associations between STI screening intensity and increases in minimum inhibitory concentrations for certain antimicrobials. Not all positive associations were statistically significant and the associations found to be statistically significant varied between the different analyses. Further studies are therefore required to assess if there is a causal relationship between the intensity of STI screening in MSM and gonococcal resistance.

# Peer review: Referee response for version 2

**DOI:** 10.5256/f1000research.17953.r38823

**Published:** 2018-10-12

**Authors:** Edward Goldstein

**Affiliations:** 1 Center for Communicable Disease Dynamics (CCDD), Department of Epidemiology, Harvard T.H. Chan School of Public Health, Boston, MA, USA

The reviewer wishes to thank the author for the revisions and the additional analyses performed. It appears to the reviewer that the results stated in the the Abstract and the results of the additional analyses in the response to the earlier reviewer comments are not fully compatible.

 The main finding of the paper, stated in the Abstract is: We found positive associations between STI screening intensity and increases in minimum inhibitory concentrations [between 2007-2013] for cefixime and azithromycin, but not ceftriaxone.”

 At the same time, according to the author’s response:

 1. “we have assessed Spearman's correlation between percent reporting screening for any STI in the previous 12 months and MIC for the three antimicrobials in the following year (2012):

 Azithromycin: Rho 0.45; P=0.141

 Cefixime: Rho 0.31; P=0.325

 Ceftriaxone: Rho=0.64; P=0.026”

 Thus the only significant relation between screening levels during the previous year and MIC is for ceftriaxone, unlike the relation between screening and increases in MIC reported in the Abstract.

 2.  We also assessed Spearman's correlation between percent reporting screening for any STI in the previous 12 months (2011 survey) and the fold change in geometric mean MIC for the three antimicrobials between

 a) 2005 and 2013:

 Azithromycin: Rho 0.77; P=0.003

 Cefixime: Rho 0.82; P=0.001

 Ceftriaxone: Rho=0.61; P=0.034

 Thus the relation between screening and increases in MIC between 2005-2013 is significant for all the three antibiotic classes, unlike the case of the increases in MIC between 2007-2013 reported in the Abstract.

 b) 2009 and 2013:

 Azithromycin: Rho 0.33; P=0.299

 Cefixime: Rho -0.50; P=0.100

 Ceftriaxone: Rho=0.58; P=0.047

 Thus the relation between screening and increases in MIC between 2009-2013 is significant only for ceftriaxone, which is exactly the opposite of the case of the increases in MIC between 2007-2013 reported in the Abstract.

 Overall, the findings stated in the Abstract reflect the results of some of the analyses, while other analyses yield different results. The author needs to think of a way for framing the results to accommodate all the findings.

I have read this submission. I believe that I have an appropriate level of expertise to state that I do not consider it to be of an acceptable scientific standard, for reasons outlined above.

# Response

**Published:** 2018-10-12

**Authors:** Chris Kenyon

**Affiliations:** Institute of Tropical Medicine, Antwerp, Belgium

Reply:

 Thank you for pointing this inconsistency out. To optimally deal with this I have added the results comparing the 2011 STI screening prevalence with the 2012 MICs to the methods, results and discussion sections.

**The sentence referring to the results in the abstract has been reworded to the following:**

 We found positive associations between STI screening intensity and increases in minimum inhibitory concentrations for cefixime and azithromycin, but not ceftriaxone when using change in city geometric mean *N. gonorrhoeae* MIC between 2005 and 2013.

**The following text has been added to the methods section:**

 The geometric mean MIC was calculated as the *n*th root of the product of *n* MIC values. Spearman's correlation was used to assess if there was an association between (1) the prevalence of STI testing in each survey and the increase in geometric mean MIC of cefixime, ceftriaxone and azithromycin in *N. gonorrhoeae* between 2005 and 2013 and (2) the percent reporting screening for any STI in the 2011 survey and geometric mean MIC for the three antimicrobials in the following year.

**The following text has been added to the results section:**

 Spearman's correlation between percent reporting screening for any STI in 2011 and geometric mean MIC for the three antimicrobials in the following year revealed a positive association for ceftriaxone (rho=0.64, p=0.026) but not for azithromycin (rho=0.45, p=0.141) or cefixime (rho=0.31, p=0.325).

**The new results are then discussed in the discussion as follows:**

 The proportion testing for bacterial STIs was associated with an increase of MIC for cefixime and azithromycin but not ceftriaxone over the time period 2005 to 2013. The correlations between percent screening in 2011 and MIC in the following year were different in that the only significant association was for ceftriaxone. Of note all six correlations between percent screening and MIC were positive. The difference between the two types of analyses related to the strengths of the associations.

# Peer review: Referee response for version 2

**DOI:** 10.5256/f1000research.17953.r38824

**Published:** 2018-10-01

**Authors:** Ellen Stobberingh

**Affiliations:** 1 Care And Public Health Research Institute (CAPHRI), Maastricht University Medical Centre (MUMC) , Maastricht, The Netherlands

No further comments

I have read this submission. I believe that I have an appropriate level of expertise to confirm that it is of an acceptable scientific standard.

# Peer review: Referee response for version 1

**DOI:** 10.5256/f1000research.16982.r38243

**Published:** 2018-09-24

**Authors:** Ellen Stobberingh

**Affiliations:** 1 Care And Public Health Research Institute (CAPHRI), Maastricht University Medical Centre (MUMC) , Maastricht, The Netherlands

The manuscript describes the association between STI screening and antimicrobial resistance development of N. gonorrhoeae. Although of interest there are several questions which need to be answered:

**Material and Methods:**

- The number of participants reduced over time: 10,030 in 2005 and 8,012 in 2011. Also there was a difference in participation rate in the different cities.  
  What is the influence of the decreased participation rate over time and the variation in the participation in the different cities on the interpretation of the data? 
- In 2008 (only syphilis) and in the two other years in addition N. gonorrhoeae, other STI or Chlamydia were tested. What was the reason for this difference?
- How many N. gonorrhoeae isolates were included in the different years?
- Was the microbiological method to isolate, identify and antibiotic susceptibility testing over testing similar over time? Which method was used for susceptibility testing?
- Please provide range and GM MIC values of the three antibiotica testing in the different years,

**Figure 1:**

- The horizontal axis mentioned an increase in MIC. What was the reference MIC?

**Dicussion:**

- Was there a change in therapy over time among the participants. All three antibiotica are mentioned in the guidelines, but is there information concerning the therapy prescribed over time in the different cities / participants?

I have read this submission. I believe that I have an appropriate level of expertise to confirm that it is of an acceptable scientific standard, however I have significant reservations, as outlined above.

# Response

**Published:** 2018-09-25

**Authors:** Chris Kenyon

**Affiliations:** Institute of Tropical Medicine, Antwerp, Belgium

The manuscript describes the association between STI screening and antimicrobial resistance development of N. gonorrhoeae. Although of interest there are several questions which need to be answered:

**Material and Methods:**

- The number of participants reduced over time: 10,030 in 2005 and 8,012 in 2011. Also there was a difference in participation rate in the different cities.  What is the influence of the decreased participation rate over time and the variation in the participation in the different cities on the interpretation of the data? 

**Reply:**

**Indeed both the decline in the absolute number of participants and the variation by city could introduce biases. This additional limitation has been added to the discussion.**

- In 2008 (only syphilis) and in the two other years in addition N. gonorrhoeae, other STI or Chlamydia were tested. What was the reason for this difference?

**Reply:**

**These differences reflect differences in the questions asked in the various surveys. In 2008, for example,** **respondents were asked if they had been tested for syphilis in the preceding 12 months, whereas in the 2011 survey they were asked if they had been tested for gonorrhea, chlamydia or syphilis in the previous 12 months. This is made clear in the methods section.**

- How many N. gonorrhoeae isolates were included in the different years?

**Reply:**

**Between 2005 and 2013, 44,144 isolates were tested. The report does not detail the breakdown of number tested by year.**

- Was the microbiological method to isolate, identify and antibiotic susceptibility testing over testing similar over time? Which method was used for susceptibility testing?

**Reply:**

**There were small changes in the laboratory protocol used. The protocol used and these changes are detailed in the Kirkcaldy et al report as follows** **[1]** **:**

**"Gonococcal isolates collected at each sentinel**

**clinic are subcultured at the clinic’s local public health laboratory on supplemented chocolate medium and frozen in Trypticase soy broth containing 20% glycerol.**

**Isolates are shipped monthly to one of the regional**

**reference laboratories, where they are tested for Beta-lactamase production and susceptibility to azithromycin, penicillin, tetracycline, ciprofloxacin, spectinomycin, cefixime, and ceftriaxone using the agar dilution method.**

**Isolates were inoculated on Difco GC medium base supplemented with**

**1% IsoVitaleX enrichment (Becton-Dickinson Diagnostic Systems,**

**Sparks, MD). During 2005 to 2007, the lowest azithromycin concentration**

**tested was 0.008 ug/ml; this increased to 0.03 ug/ml in 2008.**

**The routine testing range for azithromycin extended to 16.0 ug/ml during**

**2005 to 2013. Laboratories were asked to conduct agar dilution testing to identify an endpoint for isolates with an MIC of 16.0ug/ml on the initial**

**testing run. Testing to an endpoint was not conducted on three isolates**

**collected during 2005 to 2013. In the absence of Clinical and Laboratory**

**Standards Institute (CLSI) breakpoints for gonococcal azithromycin susceptibility or resistance, we defined reduced azithromycin susceptibility for this analysis as an MIC of  2.0 ug/ml. Quality assurance processes are described in detail in the GISP protocol.**

**To ensure accuracy and reproducibility of antimicrobial susceptibility**

**results from the regional reference laboratories, a set of seven control N. gonorrhoeae strains with known MICs of various antimicrobials are included with each susceptibility run. In addition, reference laboratories test a CDC-provided panel of 15 unidentified strains twice yearly to compare results and ensure consistency among laboratories. The results obtained from the testing of control strains and CDC-provided panels are used for internal quality assurance."**

 

- Please provide range and GM MIC values of the three antibiotica testing in the different years,

**Reply:**

**This data is not provided in the paper that reports the GISP results** **[1]** **. In the online Technical Appendix file of this paper the following data is however reported:**

https://doi.org/10.3201/eid2310.170488

**The geometric MIC of cefixime (Table 1), ceftriaxone (Table 2) and azithromycin (Table 3) by site and year. Table 8 presents the median and interdecile range of the 3 antimicrobials by site.**

**Figure 1:**

- The horizontal axis mentioned an increase in MIC. What was the reference MIC?

**Reply:**

**The horizontal axis is the change in MIC between 2007 and 2013.**

**Discussion:**

- Was there a change in therapy over time among the participants. All three antibiotica are mentioned in the guidelines, but is there information concerning the therapy prescribed over time in the different cities / participants?

**Reply:**

**This information is not provided in the report.**

**References:**

1.         Kirkcaldy RD, Bartoces MG, Soge OO, Riedel S, Kubin G, Del Rio C, et al. Antimicrobial Drug Prescription and Neisseria gonorrhoeae Susceptibility, United States, 2005-2013. Emerg Infect Dis. 2017;23(10):1657-63. Epub 2017/09/21. doi: 10.3201/eid2310.170488. PubMed PMID: 28930001; PubMed Central PMCID: PMCPMC5621530

# Peer review: Referee response for version 1

**DOI:** 10.5256/f1000research.16982.r37099

**Published:** 2018-08-20

**Authors:** Edward Goldstein

**Affiliations:** 1 Center for Communicable Disease Dynamics (CCDD), Department of Epidemiology, Harvard T.H. Chan School of Public Health, Boston, MA, USA

The paper under review studies the association between the frequency of STI screening in MSM and changes in geometric mean MICs for certain antibiotics in *N. gonorrhoeae* samples in select US cities. An important potential source of bias in such analysis could be reverse causality, namely places with higher prevalence of drug resistance for gonorrhea, as well as syphilis in MSM may initiate more STI screening efforts. Below are some questions/suggestions to the author.

 1. Please define geometric mean MIC. Also, since one refers to geometric mean, it might be more reasonable to examine fold changes (increase or decreases) in geometric mean MIC (or logarithm of thereof) in the correlation analysis.

 2. Nine cities reported STI screening information in 2005 (Figure 1), and 12 cities reported STI screening information in 2013. The reviewer would suggest to correlate the frequency of STI screening in 2013 with

  

- Geometric mean MICs in 2013
- (Fold) change in geometric mean MICs between 2009 and 2013
- (Fold) change in geometric mean MICs between 2005 and 2013

 

 For the 2005 screening frequencies, it would be interesting to correlate them with geometric mean MICs (in 2013, and possibly 2005), and not only changes in thereof.

 3. In ref. 3, no association was found between rates of antibiotic prescribing and geometric mean MICs. Perhaps the results of the paper under review are affected by reverse causality, namely places with higher prevalence of drug resistance for gonorrhea, as well as syphilis in MSM may initiate more STI screening efforts. For example, the California sites had both high geometric mean MIC for ciprofloxacin in 2005 (ref. 3), and high frequency of screening (see also ^1^). Currently, this possibility is not mentioned in the Discussion. The reviewer was also wondering if there is a correlation between the fold change in screening frequency and the fold change in geometric mean MICs between 2005 and 2013.

 4. Are other variables in NHBS-MSM surveys (possibly rate of change of sexual partners, or a number of recent sexual partners) correlated with geometric mean MICs/changes in thereof?

 5. In the Discussion, it is mentioned that “the sample size was small (9 or 12)”. Currently, only 9 cities are used in the correlation analysis, as far as the reviewer could see.

I have read this submission. I believe that I have an appropriate level of expertise to confirm that it is of an acceptable scientific standard, however I have significant reservations, as outlined above.

## References

[1] Centers for Disease Control and Prevention CDC: Increases in fluoroquinolone-resistant Neisseria gonorrhoeae among men who have sex with men--United States, 2003, and revised recommendations for gonorrhea treatment, 2004. *MMWR Morb Mortal Wkly Rep*.2004;53(16) :335-8 15123985

# Response

**Published:** 2018-09-25

**Authors:** Chris Kenyon

**Affiliations:** Institute of Tropical Medicine, Antwerp, Belgium

The paper under review studies the association between the frequency of STI screening in MSM and changes in geometric mean MICs for certain antibiotics in *N. gonorrhoeae* samples in select US cities. An important potential source of bias in such analysis could be reverse causality, namely places with higher prevalence of drug resistance for gonorrhea, as well as syphilis in MSM may initiate more STI screening efforts. Below are some questions/suggestions to the author.

1. Please define geometric mean MIC. Also, since one refers to geometric mean, it might be more reasonable to examine fold changes (increase or decreases) in geometric mean MIC (or logarithm of thereof) in the correlation analysis.

**Reply:**

**The geometric mean MIC has now been defined in the methods section.**

2. Nine cities reported STI screening information in 2005 (Figure 1), and 12 cities reported STI screening information in 2013. The reviewer would suggest to correlate the frequency of STI screening in 2013 with

 

- Geometric mean MICs in 2013
- (Fold) change in geometric mean MICs between 2009 and 2013
- (Fold) change in geometric mean MICs between 2005 and 2013

 

For the 2005 screening frequencies, it would be interesting to correlate them with geometric mean MICs (in 2013, and possibly 2005), and not only changes in thereof.

**Reply:**

**The third NHBS survey was in 2011 (not 2013) and thus we have assessed Spearman's correlation between percent reporting screening for any STI in the previous 12 months and MIC for the three antimicrobials in the following year (2012):**

**Azithromycin: Rho 0.45; P=0.141**

**Cefixime: Rho 0.31; P=0.325**

**Ceftriaxone: Rho=0.64; P=0.026**

**We also assessed Spearman's correlation between percent reporting screening for any STI in the previous 12 months (2011 survey) and the fold change in geometric mean MIC for the three antimicrobials between**

**a) 2005 and 2013:**

**Azithromycin: Rho 0.77; P=0.003**

**Cefixime: Rho 0.82; P=0.001**

**Ceftriaxone: Rho=0.61; P=0.034**

**b) 2009 and 2013:**

**Azithromycin: Rho 0.33; P=0.299**

**Cefixime: Rho -0.50; P=0.100**

**Ceftriaxone: Rho=0.58; P=0.047**

3. In ref. 3, no association was found between rates of antibiotic prescribing and geometric mean MICs. Perhaps the results of the paper under review are affected by reverse causality, namely places with higher prevalence of drug resistance for gonorrhea, as well as syphilis in MSM may initiate more STI screening efforts. For example, the California sites had both high geometric mean MIC for ciprofloxacin in 2005 (ref. 3), and high frequency of screening (see also ^1^). Currently, this possibility is not mentioned in the Discussion. The reviewer was also wondering if there is a correlation between the fold change in screening frequency and the fold change in geometric mean MICs between 2005 and 2013.

**Reply:**

**Thank you for this interesting suggestion which has been added to the discussion.**

4. Are other variables in NHBS-MSM surveys (possibly rate of change of sexual partners, or a number of recent sexual partners) correlated with geometric mean MICs/changes in thereof?

**Reply:**

**Whilst we agree this would be interesting to investigate, we did not assess these correlations in this small study.**

5. In the Discussion, it is mentioned that “the sample size was small (9 or 12)”. Currently, only 9 cities are used in the correlation analysis, as far as the reviewer could see.

**Reply:**

**The sample size for 2005 was 9 and therefore in Figure 1, where one of the variables is from 2005, only 9 data points can be used. There were however 12 data points for the other 2 surveys and these were used in the analyses using these surveys.**

**References:**

1.         Kirkcaldy RD, Bartoces MG, Soge OO, Riedel S, Kubin G, Del Rio C, et al. Antimicrobial Drug Prescription and Neisseria gonorrhoeae Susceptibility, United States, 2005-2013. Emerg Infect Dis. 2017;23(10):1657-63. Epub 2017/09/21. doi: 10.3201/eid2310.170488. PubMed PMID: 28930001; PubMed Central PMCID: PMCPMC5621530.
